# Efficient p‐Type Doping of Tin Halide Perovskite via Sequential Diffusion for Thermoelectrics

**DOI:** 10.1002/smsc.202200004

**Published:** 2022-03-06

**Authors:** Ruisi Chen, Yajie Yan, Junhui Tang, Huarong Zeng, Qin Yao, Lidong Chen, Ziqi Liang

**Affiliations:** ^1^ Department of Materials Science Fudan University Shanghai 200433 China; ^2^ State Key Laboratory of High-Performance Ceramics and Superfine Microstructure Shanghai Institute of Ceramics Chinese Academy of Sciences Shanghai 200050 China

**Keywords:** air doping, diffusion doping, electrical conductivity/thermoelectrics, F_4_TCNQ, FASnI_3_

## Abstract

Metal halide perovskites (MHPs) hold great potential in thermoelectric (TE) applications, thanks to their regular and soft lattice in nature. However, the poor electrical conductivity caused by low charge carrier density (<10^14^ cm^−3^ for lead‐based MHPs) strongly impedes its TE development. In this scenario, tin halide perovskites (THPs) emerge as promising TE candidates owing to their high background hole densities (>10^19^ cm^−3^). However, further electrical doping remains challenging, originating from the limited capability of accommodating heterogeneous dopants and the heavy compensation in THPs. Herein, a novel diffusion‐mediated doping approach is demonstrated to prominently increase the p‐type doping level of THPs by a sequence of air exposure and 2,3,5,6‐tetrafluoro‐7,7,8,8‐tetracyanoquinodimethane (F_4_TCNQ) surface treatments. In paradigm photovoltaic THP materials—CH(NH_2_)_2_SnI_3_ (namely FASnI_3_), the electrical conductivity is dramatically increased by 300× from 0.06 to 18 S cm^−1^ in thin films, leading to a remarkable enhancement of power factor by 25× up to 53 μW m^−1^ K^−2^. In contrast, only a slight variation of thermal conductivity is observed after F_4_TCNQ deposition, which is in accordance with the increase in electrical conductivity, indicating that the lattice structures of FASnI_3_ remain intact after doping. This study paves an illuminating way to ameliorate TE properties in halide perovskites.

## Graphical abstract

A sequential diffusion doping strategy of air exposure and then F_4_TCNQ surface treatment is demonstrated in CH(NH_2_)_2_SnI_3_ films to achieve a remarkably increased electrical conductivity up to 18 S cm^−1^, leading to an impressive thermoelectric power factor of 53 μW m^−1^ K^−2^. Such diffusion doping enables effective interactions between heterogenous dopants and CH(NH_2_)_2_SnI_3_ lattices, while revealing the underlying distinct doping mechanisms.

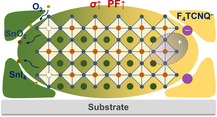

## 1 Introduction

Rapid development has been witnessed in the past decade on metal halide perovskites (MHPs), particularly in the field of optoelectronics on account of their uniquely high defect tolerance, long exciton diffusion length, bandgap tunability, and low‐cost solution processability.^[^ ^1^, ^2^, ^3^, ^4^, ^5^ ^]^ The chemical formula of MHPs is denoted as ABX_3_ where A represents the monovalent organic or inorganic cation, B is a metal cation such as Sn^2+^ or Pb^2+^, and X is a halide anion.^[^ ^6^ ^]^ MHPs structurally consist of the corner‐sharing metal [BX_6_]^4-^ octahedra and the interstitial A‐site cations, which jointly endow them with intrinsically high carrier mobility (*μ*) and attenuated heat transport, resulting in electron‐crystal‐like and phonon‐glass‐like behavior, respectively, thereby holding broad prospects for thermoelectric (TE) applications.^[^ ^7^, ^8^, ^9^ ^]^ Typically, the performance of TE materials is determined by the mutually coupled characteristics of electrical conductivity (*σ*, S cm^−1^), Seebeck coefficient (*S*, μV K^−1^), and thermal conductivity (*κ*, W m^−1 ^K^−1^), which collectively contribute to the dimensionless figure of merit (denoted as *zT*) in the form of *S* ^*2*^ *σT/κ*, where *T* is the absolute temperature and *S* ^*2*^ *σ* is also defined as power factor (PF, μW m^−1 ^K^−2^).^[^ ^10^ ^]^

Although the electrical and thermal characteristics of lead–based MHPs have been widely studied, the TE performance is still far from satisfactory because of their intrinsically low *σ*.^[^ ^7^ ^]^ Tin halide perovskites (THPs) recently stood out with an inherently higher carrier concentration than their lead counterparts due to the characteristic self‐doping effect, which is conducive to superior electrical conductivity and hence renders them considerably favorable for TE applications.^[^ ^11^, ^12^ ^]^ However, the carrier density of THPs is still constrained by the limited capability of accommodating heterogeneous dopants and the heavy compensation from intrinsic defects, both of which severely undermine their TE performances. The studies of THP‐based TE materials are rarely reported and have just come to burgeon in recent years,^[^ ^13^, ^14^, ^15^, ^16^ ^]^ most of which were centered upon diversified doping strategies. Among them, all‐inorganic CsSnX_3_ perovskites have attracted enormous attention, thanks to their relatively high *σ*.^[^ ^13^ ^]^ To exemplify it, single‐crystal CsSnI_3_ nanowires yielded a superior *σ* of 282 S cm^−1^ without optimization.^[^ ^17^ ^]^ A further study reported an encouraging increase in *σ* by one order of magnitude via air doping the thermally vapor‐deposited CsSnI_3_ films with a chlorine‐rich capping layer.^[^ ^18^ ^]^ Nevertheless, these fabrication methods of all‐inorganic perovskites are costly and complex, which would be unsuitable for scalable production. Meanwhile, inorganic halide perovskites were revealed to possess higher *κ* than their organic/inorganic counterparts, which is mostly ascribed to the organic cation dynamic disorder.^[^ ^19^ ^]^ Early studies were focused on CH_3_NH_3_SnI_3_ (namely, MASnI_3_)‐based crystals^[^ ^20^, ^21^ ^]^ and showed the extrinsic doping of Sn^4+^ ions as an efficient method to increase the carrier concentration by fivefolds yet failed to achieve a satisfactory *σ* in THPs. In addition, the thermal instability of the MA component impaired long‐term stable operation. More recently, another benchmark family of hybrid THPs—CH(NH_2_)_2_SnI_3_ (i.e., FASnI_3_), which delivered the highest photovoltaic efficiency in THP‐based solar cells,^[^ ^22^, ^23^, ^24^, ^25^, ^26^ ^]^ also emerged as promising TE candidates. For instance, Gong et al. mixed a classic electron acceptor, 2,3,5,6‐tetrafluoro‐7,7,8,8‐tetracyanoquinodimethane (F_4_TCNQ), into the FASnI_3_ precursor solution and reported a noticeable improvement of *σ* up to 13.65 S cm^−1^; however, the doping level is still constrained because the insufficient dopants were incorporated purposely to ensure the desired film morphology.^[^ ^27^ ^]^ Although the aforementioned strategies have manifested as direct avenues to elevating *σ* of THPs, a lack of in‐depth understanding of interactions between dopants and perovskite lattices results in the uncontrollable and restrained doping level along with poor reproducibility across research laboratories.

In this study, we demonstrate for the first time an effective and facile surface treatment of combined air oxidation and F_4_TCNQ diffusion to significantly enhance the doping efficiency in FASnI_3_ films while enabling to unveil their distinct roles in THPs. First, we investigate the oxidation‐assisted doping process of Sn^2+^ in the FASnI_3_ film and confirm its favorable impacts by exhibiting a 50× increase in *σ*. Then, it is validated that the increased *σ* accounts for the major part of PF improvements, which highlight the priority of *σ* modulation to *S* when improving the PF of THPs. To prevent FASnI_3_ from the over air exposure, which leads to the lattice decomposition and hence worse performance, F_4_TCNQ is further exploited by surface deposition and then diffuses into the THP bulk, as verified by the time‐of‐flight secondary‐ion mass spectrometry (TOF‐SIMS). Surprisingly, it is observed that F_4_TCNQ realizes a superimposed p‐doping effect upon the as‐oxidized thin film. Joint analysis of Fourier‐transform infrared (FTIR) and X‐ray photoelectron spectroscopy (XPS) distinguishes the doping mechanisms of F_4_TCNQ and oxidation by charge and atomic transfer, respectively, which underpins the origin of the accumulated doping effect in FASnI_3_. Consequently, a striking increase in *σ* by ×300 is obtained, which leads to an outstanding PF of 53 μW m^−1^ K^−2^, accompanied also by remarkably enhanced electrical stability.

## 2 Results and Discussion

**Figure 1:**
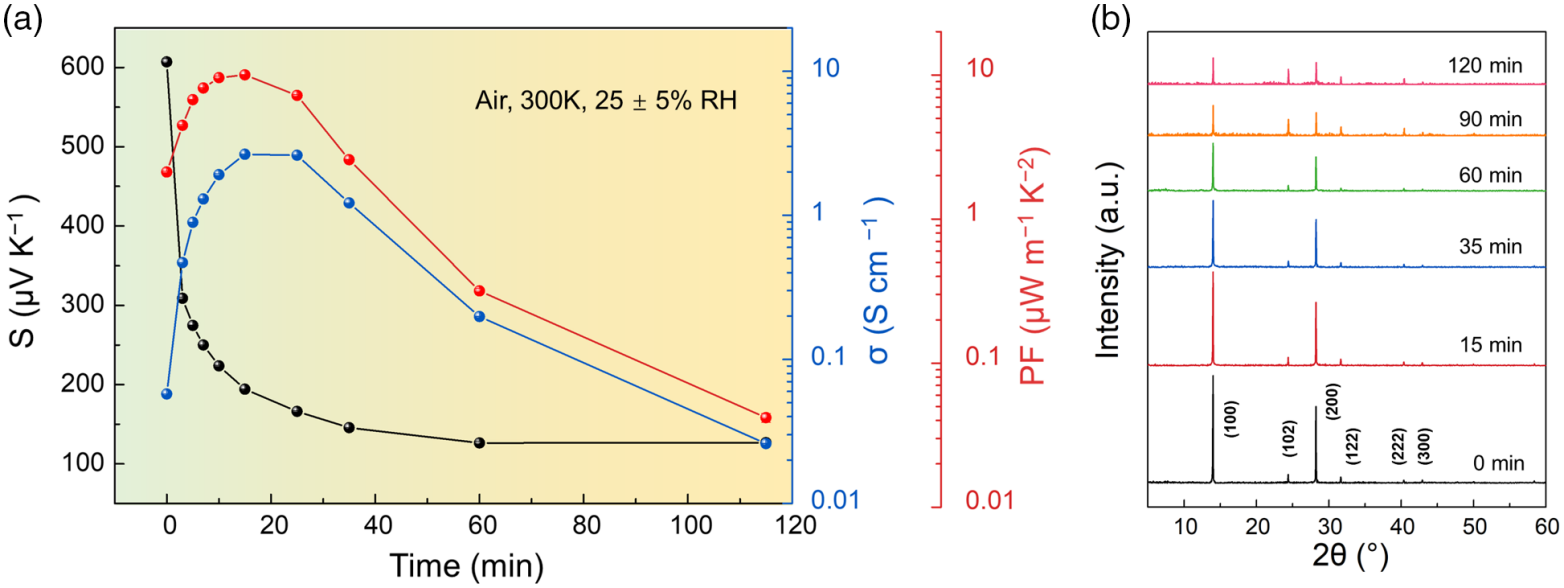
a) Electrical characteristics of FASnI_3_ thin film as a function of air exposure time. b) XRD patterns of FASnI_3_ thin film in air with lattice plane indices.

The FASnI_3_ thin films were fabricated by the facile spin‐coating method (refer to Experimental Section for details) with a thickness of ≈250 nm, which is comparable with previous literatures.^[^ ^27^, ^28^ ^]^ Time‐dependent electrical measurements were conducted to explore the air‐doping effect of FASnI_3_ thin film. As shown in **Figure** **1**a, when the thin film was exposed to air, *σ* is increased tremendously by 50× in the first 15 min as a result of p‐type doping by O_2_, showing a peak value of 2.6 S cm^−1^ at 15 min, which is presumably attributed to the dramatically enhanced carrier concentration. Further air exposure however leads to deteriorated electrical transport owing to the decomposition of the perovskite lattices. On the other hand, a sharp decrease is observed in *S* from 607 to ≈126 μV K^−1^ in the first 60 min and then *S* tends to be steady with air exposure due to the gradually saturated carrier concentration. Compared with the variation of *σ*, the change of *S* is too flattened to play a dominant role in determining the PF. Consequently, the trend of the time‐dependent PF is similar to *σ*, suggesting that the optimized PF can be achieved by direct control of the oxidation level, which can be estimated from *σ*. As a result, the highest PF of 10 μW m^−1^ K^−2^ is obtained upon air exposure for 15 min. Moreover, it is reported that the degradation of the perovskite lattice is also in intimate correlation with ambient humidity.^[^ ^29^, ^30^ ^]^ As shown in Table S1 in the Supporting Information (SI), we tested the optimal oxidation time and highest *σ* for FASnI_3_ films at room temperature with different relative humidities (RHs) of 20% and 65%. At a higher RH, the time required to achieve the peak *σ* is shortened, while the *σ* value is decreased from ≈2.50 to ≈1.34 S cm^−1^, presumably attributable to the moisture‐induced structure decomposition especially in the presence of oxygen.

To characterize the air stability of FASnI_3_ film, X‐ray diffraction (XRD) was used to probe the perovskite crystal structure. Figure 1b displays the time‐dependent XRD patterns of FASnI_3_ thin films in air, which are consistent with previous studies.^[^ ^28^, ^31^ ^]^ The peak intensity declines, while the full‐width‐half‐maximum (FWHM) of peaks is raised without additional signals during 120 min of aging, indicating that the structural components of FASnI_3_ remain relatively stable without the generation of obvious impurity phases, in spite of slightly decreased crystallinity. During the thin‐film fabrication process, SnF_2_ additive was added into the perovskite precursor solution to promote the crystallization of FASnI_3_ while acting as an antioxidant to stabilize the perovskite bulk phase against air exposure.^[^ ^32^ ^]^ As the reaction with oxygen originates from the surface, SnF_2_ shows a merit of reducing the oxidation rate to better tune the doping level.

**Figure 2:**
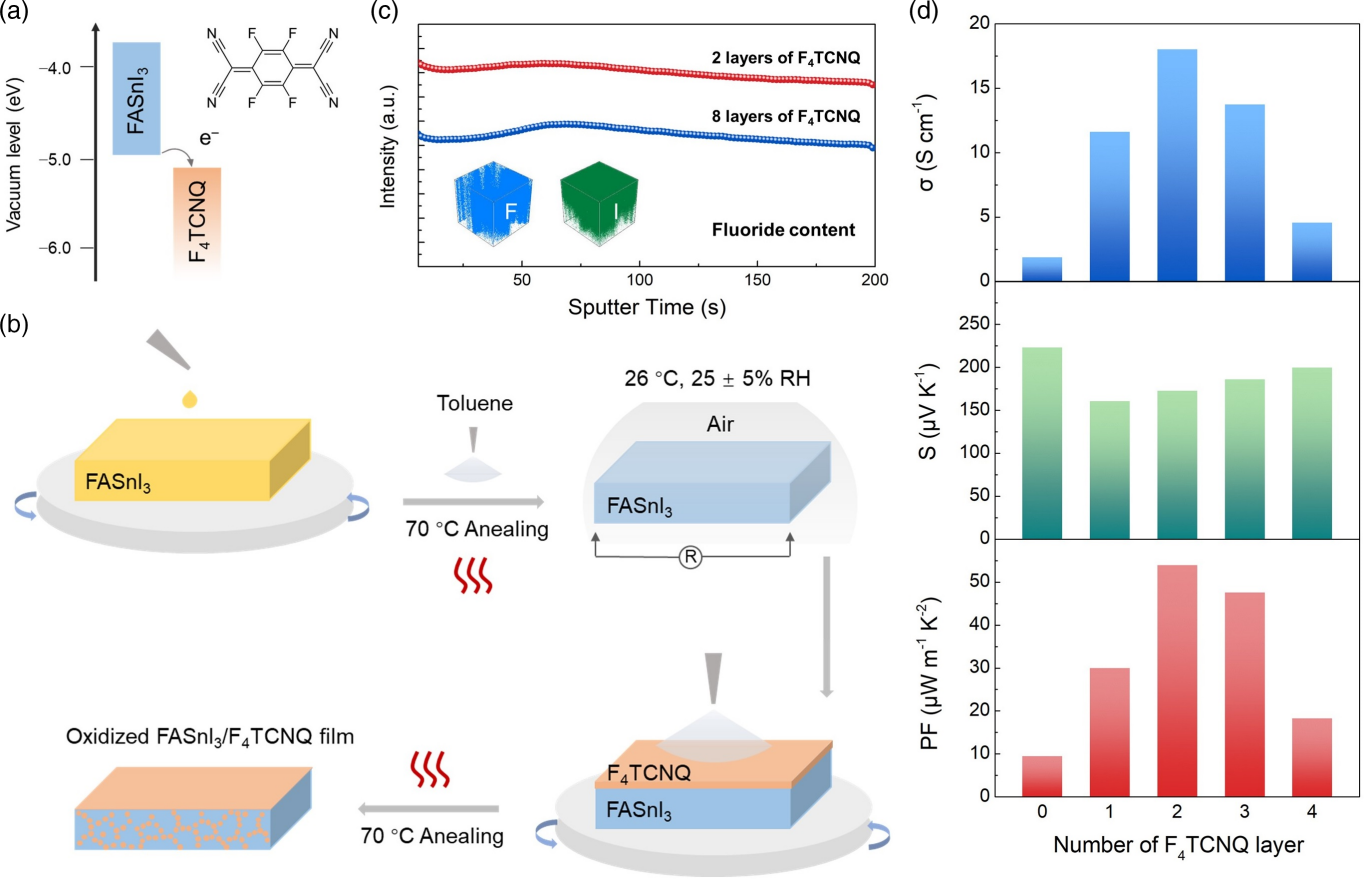
a) Schematic energy level diagram of FASnI_3_ and F_4_TCNQ. b) Illustrative thin‐film fabrication process during which F_4_TCNQ molecules enter the film by solid diffusion. c) TOF‐SIMS spectra of the film deposited with two and eight layers of F_4_TCNQ, showing a variation of F‐element content at different depths. The inset shows the 3D render overlay of F^−^ and I^−^ in FASnI_3_ films after deposition of two layers of F_4_TCNQ. d) Summary of electrical characteristics of FASnI_3_/F_4_TCNQ films with various numbers of F_4_TCNQ layer.

Nevertheless, the oxidation rate of the perovskite film varies with the environmental effects such as light illumination, temperature, humidity, and so on, rendering it difficult to attain an optimal doping level precisely. Moreover, the lattice degradation caused by the excessive O_2_ doping impairs the stability of electrical characteristics, as shown in Figure 1, which will be discussed later. To tackle these issues, F_4_TCNQ, which is one of the most widely adopted and effective p‐type dopants due to its strong electron‐withdrawing ability and the extended π‐system, was exploited to further p‐dope the oxidized perovskite. As shown in **Figure** **2**a, F_4_TCNQ possesses a deep lowest unoccupied molecular orbital (LUMO) energy level (−5.2 eV) that is energetically in the vicinity of the highest occupied molecular orbital (HOMO) energy level of FASnI_3_ (≈−5.0 eV) and hence doping is facilitated by electron transfer from the HOMO of FASnI_3_ to the LUMO of F_4_TCNQ.^[^ ^33^, ^34^ ^]^ To acquire the best *σ* without overexposing the thin film in air, F_4_TCNQ was immediately solution spray deposited onto the FASnI_3_ thin film that approaches the peak electrical characteristics. The detailed sequential doping process of the FASnI_3_ thin film is illustrated in Figure 2b. The as‐spin‐coated FASnI_3_ film was first transferred into air, during which the resistance of the film was continuously recorded for a rough estimate of the doping level. Next, F_4_TCNQ dissolved in chlorobenzene was spray coated on the surface of the oxidized FASnI_3_, followed by a thermal annealing process, during which F_4_TCNQ dopants tended to diffuse through the grain boundaries and subsequently into the bulk of thin film, as verified by TOF‐SIMS in Figure 2c. The F element is detected with the sputter time to signify the existence of F_4_TCNQ molecules at the different depths of the FASnI_3_ thin film that was prepared in the absence of SnF_2_ additive. Although the intensity of F_4_TCNQ is slightly decreased throughout thin films, the overall trend indicates that F_4_TCNQ molecules diffuse uniformly to realize additional p‐doping, which is also illustrated in the inset of Figure 2c when compared with the distribution of I element. More importantly, such diffusion doping from the surface enables efficient incorporation of the adequate molecular dopants into the FASnI_3_ thin films without adversely affecting perovskite crystallization during film fabrication.

Given the limited solubility of F_4_TCNQ in chlorobenzene, we fabricated the FASnI_3_/F_4_TCNQ films that were deposited with various numbers of doping layers to modulate the dopant contents and measured the electrical characteristics accordingly. As shown in Figure 2d, *σ* exhibits a sequence of positive and then negative trends as the number of F_4_TCNQ layers progressively increased from 0 to 4, which ultimately reached a maximum value of 18.0 S cm^−1^ with double layers of F_4_TCNQ. According to the TOF‐SIMS spectra in Figure 2c, excessive dopants continue to penetrate into the thin film instead of residing on the surface and thus deteriorate charge transport as they act as impurities/traps, leading to a decrease in *σ* value. In addition, F_4_TCNQ‐doped samples present a slight decrease in *S*, similar to the oxidation process shown in Figure 1a, which is ascribed to the increased carrier concentration. As a consequence, the enhancement of *σ* leads to the prominently improved PF from 10 to 53 μW m^−1^ K^−2^.

Moreover, it is demonstrated that the doping effect of F_4_TCNQ on *σ* places a superposition on that of O_2_. Based on the equation of *σ* = *nqμ* where *n* is carrier concentration and *q* is elementary charge, the carrier concentration before and after doping can be evaluated by characterizing the carrier mobility of as‐prepared films. According to the grazing‐incidence wide‐angle X‐ray scattering (GIWAXS) pattern in Figure S1 in Supporting Information, the isotropy of FASnI_3_ film allows to estimate the in‐plane mobility by the out‐of‐plane mobility. Then, the space–charge‐limited‐current (SCLC) method was adopted extract the charge mobility. As shown in Figure S1h in Supporting Information, both the electron and hole mobilities feature a slight decrease after oxygen and F_4_TCNQ treatments because of the lattice degradation and the impurities, revealing that an increase of *σ* is mainly ascribed to the incremented hole carriers with an elevated doping level.

The optical spectroscopies were also conducted to corroborate the doping level upon various air‐exposure times and F_4_TCNQ treatment. As shown in Figure S2 in Supporting Information, the optical bandgap (*E*_g_) as calculated from the UV–vis spectra of thin films exhibits a continuously increasing trend from neat ≈1.39 eV (FASnI_3_) to ≈1.47 eV (sequentially doped FASnI_3_). As no additional absorption can be observed relevant to decomposed products such as SnI_4_ (≈2.6 eV shown in Figure S3, Supporting Information) and SnO_2_ (≈4.0 eV),^[^ ^35^ ^]^ such a broadening of *E*_g_ may be attributed to either crystal refinement or Burstein−Moss effect caused by the heavy p‐doping.^[^ ^36^ ^]^ Based on the Scherrer equation, the grain sizes perpendicular to the (100) facets are determined to be no less than 769 nm in the XRD patterns without a quantum confinement effect. Consequently, this blueshift of the absorption edge with the air‐exposure time and F_4_TCNQ deposition results from the dramatically increased p‐doping level, which leads to *σ* enhancement by sequential doping, as shown in Figure 1a and 2d. Such an elevated doping level probably originates from the different interaction mechanisms of dopants. It is further revealed that either O_2_ or F_4_TCNQ doping leads to a successive optimization of *σ* based on the other treatment. Figure S4 in Supporting Information shows the electrical properties of FASnI_3_ films deposited with single‐ and dual‐layer F_4_TCNQ before and after oxidation. The lower *σ* value prior to air exposure indicates that the impact of F_4_TCNQ alone on the electrical property is also limited in comparison with the F_4_TCNQ and oxidation dual treatments. Such a phenomenon confirms the distinct doping mechanism between O_2_ and F_4_TCNQ, which will be elaborated in the following part.

**Figure 3:**
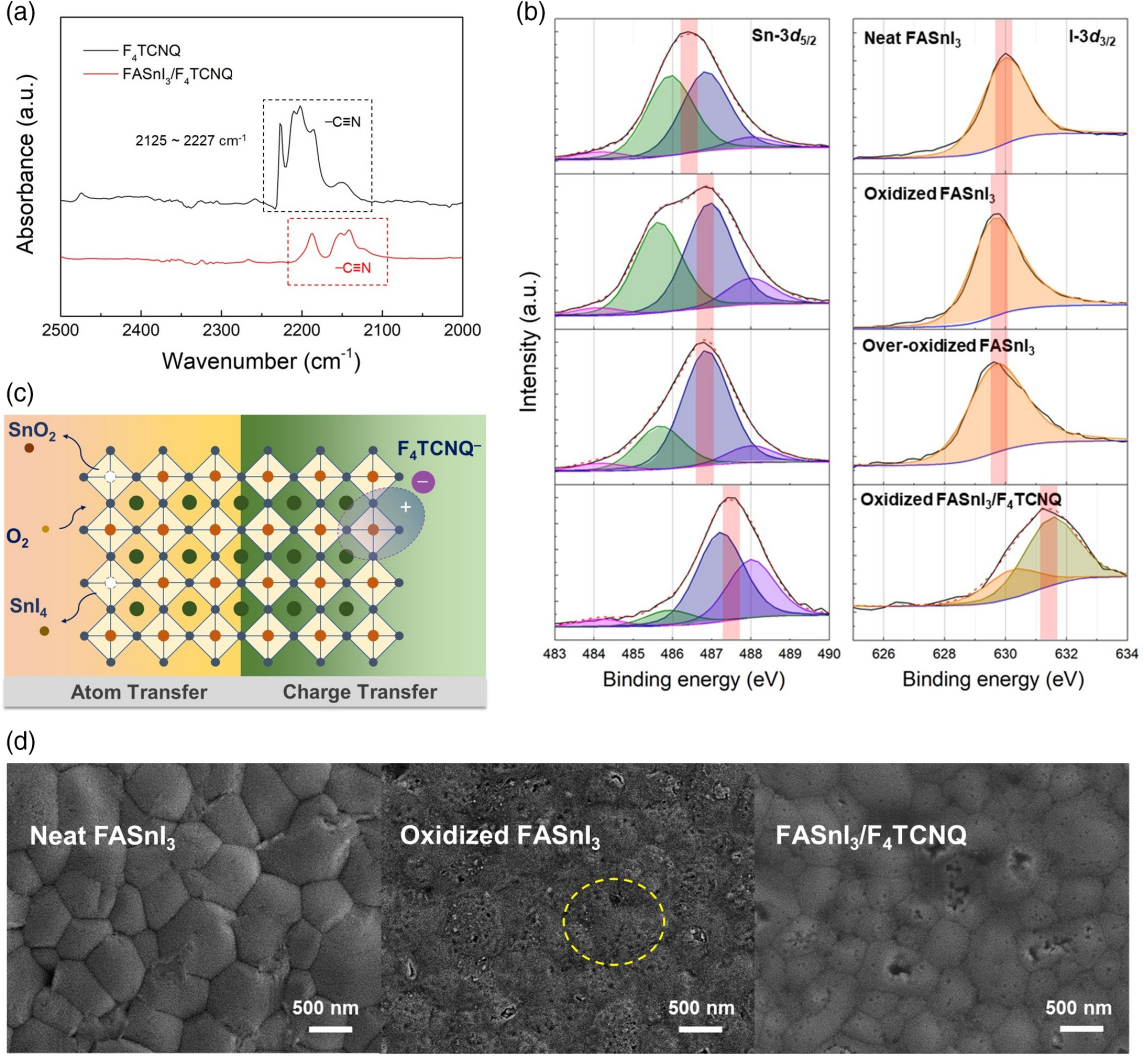
a) FTIR spectra of neat F_4_TCNQ and FASnI_3_/F_4_TCNQ films in *ν*_C≡N_ region. b) Sn 3*d*_5/2_ and I 3*d*_3/2_ XPS spectra of FASnI_3_ with various oxidation levels and after F_4_TCNQ doping. Fitted Gaussian components are also shown. c) Schematic illustration of the doping process in the case of O_2_ and F_4_TCNQ, respectively. d) FE‐SEM images of the neat, oxidized and F_4_TCNQ‐doped FASnI_3_ thin films.

To elucidate the interaction between F_4_TCNQ and perovskite lattices, the states of both dopant and host were investigated by FTIR and XPS measurements, as shown in **Figure** **3**a,b, respectively. Specific signals generated by ionized F_4_TCNQ can be detected in FTIR spectra to understand its redox properties, among which a characteristic frequency region of $\nu_{C\equiv N}$ has been widely studied because of its high sensitivity to the extent of charge transfer and the minimal spectral interferences. In the neat F_4_TCNQ film, the peak of vibration mode (b_1u_) at 2227 cm^−1^ symbolizes the neural state of F_4_TCNQ.^[^ ^37^ ^]^ After F_4_TCNQ doping of FASnI_3_, the bands of $\nu_{C\equiv N}$ shift toward lower frequencies, implying the generation of F_4_TCNQ^−^ and F_4_TCNQ^2−^ and thus verifying an occurrence of charge transfer from FASnI_3_ to F_4_TCNQ.^[^ ^38^, ^39^ ^]^ The full FTIR spectra are shown in Figure S5 (Supporting Information), where the peaks at 1395, 1341, and 1598 cm^−1^ represent the b_2u_ mode of *ν*_C−C_ as well as b_1u_ and b_2u_ modes of *ν*_C=C_, respectively. Note that the C–F signals are too weak to be identified when compared with the signals of halogenated hydrocarbons.

To distinguish the respective role of F_4_TCNQ and O_2_ during the sequential doping processes, the XPS profiles were acquired for neat, oxidized, as well as successive oxidized and F_4_TCNQ‐doped FASnI_3_ with the Sn‐3*d* and I‐3*d* binding energy spectra displayed in Figure 3b. During air exposure, both the increasing signal intensity of high‐state Sn ions and the shift of the Sn 3*d*_5/2_ peak toward higher binding energy indicate a continuous ascending valence state of Sn. Remarkably, the introduction of F_4_TCNQ layer shifts the Sn‐3*d* peak of the overoxidation sample toward higher binding energy by 0.6 eV, which substantiates the superposition doping effect of F_4_TCNQ on O_2_ as discussed earlier. On the other hand, I^−^ ions fail to interact with O_2_ molecules as no distinct peak shift is observed. However, an obvious I‐3*d* peak shift in XPS spectra appears after addition of F_4_TCNQ, implying that F_4_TCNQ also interacts with I^−^. Consequently, the difference of the two doping processes lies in the reactants, as illustrated in Figure 3c. The exposure of FASnI_3_ against oxygen follows the degradation route (1)^[^ ^40^ ^]^

(1) $${2\text{ FASnI}}_{3}{+\text{ O}}_{2}\to {\;2\text{ FAI }+\text{ SnO}}_{2}{+\text{ SnI}}_{4}$$

During this process, O_2_ scavenges two electrons from Sn^2+^ ion and therefore two holes are released into the matrix to achieve p‐type doping, as shown in Equation (2).^[^ ^41^ ^]^

(2) $${\text{Sn}}^{2+}{+\text{ O}}_{2}\to {\text{ SnO}}_{2}{+\;2h}^{+}$$

Here the surface Sn—I bonding was broken down by the O_2_ invasion, with the subsequent escaped Sn^4+^ ions from the octahedral lattice as well as the formation of SnI_4_ and SnO_2_ at the grain boundaries, which can be defined as main atom transfer process. On the other hand, only electron transfer occurs in the case of F_4_TCNQ between the [SnI_6_]^4−^ octahedra and dopants, wherein the I^−^ anions also contribute to charge transfer due to the strong electron‐withdrawing capabilities of —C ≡ N and —F radical groups. As a consequence, Sn^2+^ remains within the lattices instead of generating Sn vacancies and causing atom transfer. Such an assumption is also evidenced by the morphological variation of the samples after treatments by both dopants, as shown in the field‐emission scanning electron microscopy (FESEM) images in Figure 3d, wherein the film features pinholes (as highlighted in the yellow circles) upon air exposure, while remaining compact and homogeneous when doped by F_4_TCNQ, suggestive of the structural decomposition in the former yet the lattice integrity in the latter. Moreover, the XRD patterns of FASnI_3_ films with and without F_4_TCNQ treatment shown in Figure S6 (Supporting Information) also verify the intact crystal structure with almost no peak shift or additional peak after doping, indicating that F_4_TCNQ molecules do not penetrate into the perovskite lattice or cause structural decomposition during p‐doping.

**Figure 4:**
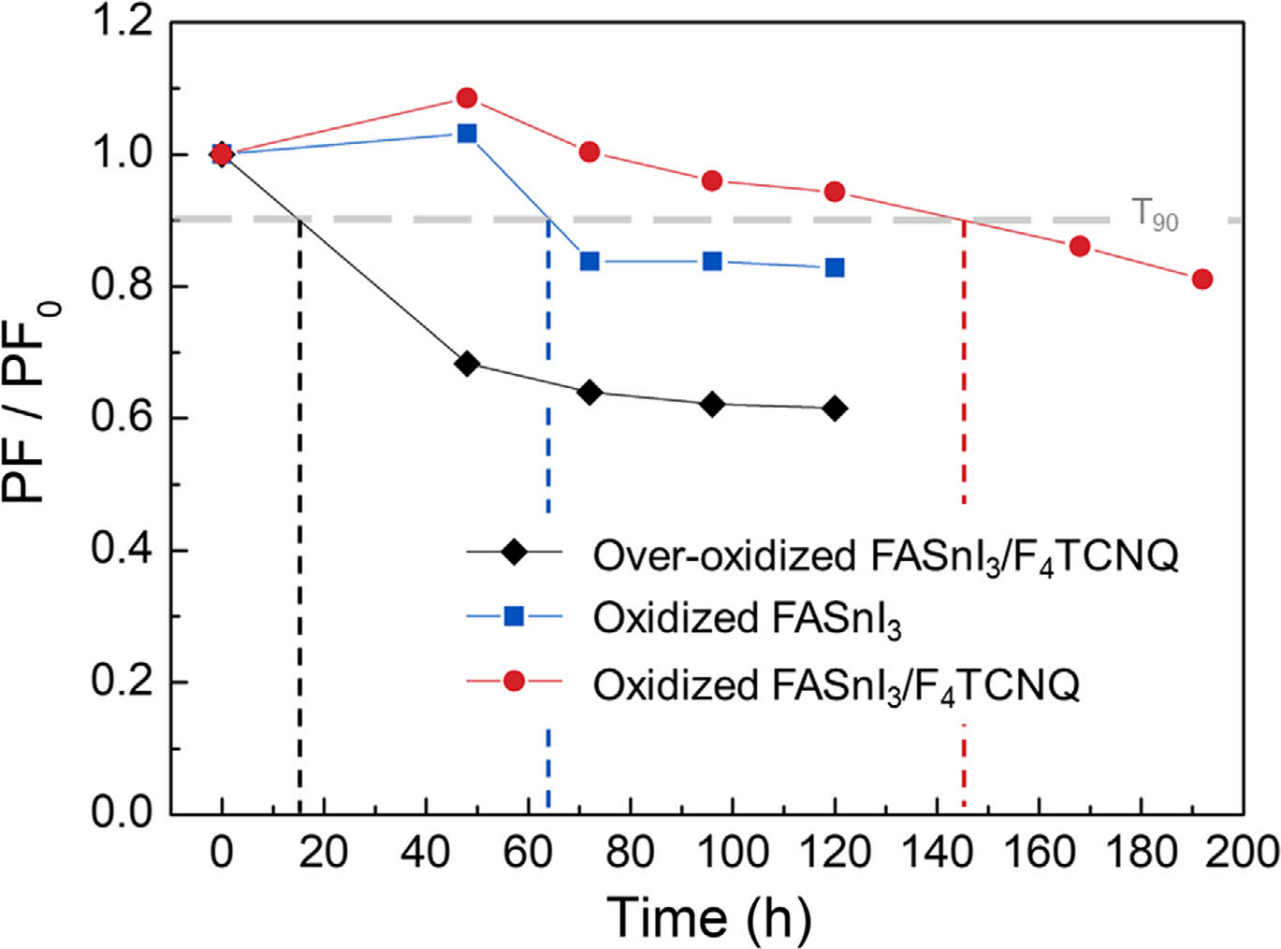
Stability of oxidized FASnI_3_, oxidized and over‐oxidized FASnI_3_/F_4_TCNQ thin films in Ar atmosphere.

As the air exposure is accompanied by the degradation of lattice structure, it retards charge transport through thin films. Thus, the time‐dependent PF variations were recorded to evaluate the durability of as‐prepared perovskite thin films at room temperature and in Ar atmosphere without encapsulation, as shown in **Figure** **4**. Here, *T*_90_ represents the time required for PF to drop down to 90% of the original sample. The over‐oxidized samples, in which the collapse of crystal structure is predominant in influencing charge transport, show the worse stability among others with a considerable PF decrease in 120 h, further highlighting the critical importance of controlling the oxidization degree of FASnI_3_. When exposed in air, a large amount of O_2_ molecules tend to adsorb on the surface of the perovskite thin film or penetrate through grain boundaries. The excessive O_2_ or the incompletely bonded O^2−^ ions continue to extract the Sn^2+^ ions from the perovskite lattice after being transferred backward to Ar atmosphere, thus leading to the additional p‐type doping during storage. This is also suggested by the slight increase in PF for FASnI_3_ and FASnI_3_/F_4_TCNQ shown in Figure 4. However, F_4_TCNQ will compete to shield the lattice from O_2_ molecules or O^2−^ ions due to the stronger electronegativity and meanwhile passivate the defects at the grain boundaries, which exert a positive effect on preventing the crystal structures from rapid degradation. Consequently, the FASnI_3_/F_4_TCNQ hybrid film delivers the best electrical stability among others.

It is generally acknowledged that MHPs possess an ultralow *κ* due to both low‐phonon group velocities and short‐phonon lifetimes.^[^ ^7^ ^]^ It is therefore essential to distinguish the diffusion doping process in this study from the other bulk doping strategies in literatures with regard to the impacts on *κ*. An accurate measurement of *κ* however affords a big hurdle for THP thin films due to the limited capability of available methods to characterize the in‐plane thermal diffusion in such nonself‐supporting thin films as well as the ambient instability of THPs as discussed earlier. Consequently, the thermal transport property of THP films has been barely studied. Moreover, it remains obscure whether the inclusion of F_4_TCNQ results in an increase in *κ*, which possibly counteracts its benefits on PF. Here we exploited scanning thermal microscopy (SThM) to investigate the effect of F_4_TCNQ doping on the *κ*_total_ of FASnI_3_. In the SThM method, an atomic force microscopy platform is used to detect the change of thermal resistance based on which the *κ*_total_ can be calculated (refer to the details in SI). The *κ*_total_ of as‐oxidized FASnI_3_ is estimated to be 0.286 ± 0.007 W m^−1 ^K^−1^ at *σ* ≈ 2.6 S cm^−1^ (room temperature), which is even lower than the reported value of MAPbI_3_ polycrystalline films (≈0.3 W m^−1 ^K^−1^).^[^ ^42^, ^43^ ^]^ Further surface diffusion of F_4_TCNQ dopants slightly increases the *κ*_total_ to 0.296 ± 0.007 W m^−1 ^K^−1^ at *σ* ≈ 18.0 S cm^−1^, which is comparable with a literature value of F_4_TCNQ bulk‐doped FASnI_3_ films at *σ* = 13.65 S cm^−1^ by the same measurement.^[^ ^27^ ^]^ Although a sixfold enhancement in *σ* is observed upon F_4_TCNQ deposition on air‐oxidized FASnI_3_, the increase in *κ*_total_ is relatively insignificant (≈0.01 W m^−1 ^K^−1^). Note that *κ*_total_ comprises electron contribution (*κ*_e_) and phonon/lattice contribution (*κ*_L_), of which *κ*_e_ follows Wiedemann–Franz's law.^[^ ^44^, ^45^ ^]^

(3) $$\kappa_{e}=L\sigma T$$

where *L* is the Lorentz number. Based on Equation (3), *κ*_e_ can be calculated as approximately 0.01 W m^−1 ^K^−1^ for the F_4_TCNQ‐doped film, for which we attribute this subtle increase in *κ*_total_ to the enhanced *σ*, suggesting that F_4_TCNQ penetration imposes negligible effects on the lattice structures of FASnI_3_ while merely residing the grain boundaries and thus leads to fairly stable *κ*_L_, which plays a decisive role in *κ*_total_. As a result, the *zT* value of the optimal F_4_TCNQ‐doped sample is estimated to be ≈0.05 at room temperature.

## 3 Conclusions

In conclusion, we have successfully demonstrated a sequential strategy by combining the oxidation‐assisted optimal doping and diffusion doping via F_4_TCNQ surface treatment to achieve continuous elevation of the doping level in FASnI_3_ thin films, leading to a dramatically increased *σ* of 18 S cm^−1^ and hence a remarkable PF of 53 μW m^−1^ K^−2^, accompanied by greatly improved electrical stability. Such a diffusion‐mediated doping strategy not only allows for the facile and noninvasive inclusion of dopants into FASnI_3_ films but also underpins the significance of the two‐step doping method with an interesting superimposed effect on elevating the doping level by distinct mechanisms. Unsatisfactorily, it is worth noting that *S* in our work is constrained by the increased carrier concentration. Most recently, high *S* and excellent thermal stability were realized in 2D tin‐based perovskites because of their quantum confinement effect and organic ligands, respectively.^[^ ^15^, ^46^ ^]^ We therefore envision that the surface doping strategy demonstrated in this work, when applied to low‐dimensional THPs, may further boost the TE performance.

## 4 Experimental Section

4.1

4.1.1

##### Chemicals and Materials

FAI (99.9%) and SnF_2_ (99.9%) were purchased from Maituowei Ltd. (China) while SnI_2_ (99.9%) was from Alfa Aesar (China). All other chemicals were purchased from J&K Scientific, Ltd. (China). All the reagents were used as received.

##### Thin‐Film Fabrication

The pristine FASnI_3_ precursor solution (1M) was prepared by mixing SnI_2_, FAI, and SnF_2_, at a stoichiometric ratio of 10:10:1 in mixed DMF:DMSO (4:1 vol%) solvent. Then, the precursor solution was stirred under room temperature at 600 rpm for 1 h. For film fabrication, the FASnI_3_ solution was first spin coated on an indium tin oxide (ITO)‐patterned glass substrate at 5000 rpm at room temperature for 60 s, during which toluene was deposited as antisolvent to accelerate the crystallization of the FASnI_3_ film. The as‐prepared films were heated at 70 °C in inert atmosphere for 20 min to remove residual solvent and then transferred into air at ambient temperature (26 °C) and at RH of 25 ± 5% with an oxygen concentration of ≈20.8%, during which the resistance of the film was continuously recorded for a rough estimate of the doping level. Next, F_4_TCNQ was dissolved in chlorobenzene solvent as saturated solution (1.5 mg mL^−1^) and subsequently spray coated on the oxidized FASnI_3_, followed by the thermal annealing process. All samples were prepared in argon (Ar) atmosphere (H_2_O < 0.1 ppm, O_2_ < 0.1 ppm) glovebox.

##### Characterization and Measurements

Electrical properties of thin films were measured at room temperature using a custom‐built apparatus according to our previous report.^[^ ^47^ ^]^ A four‐probe technique was used to measure electrical conductivity on a multimeter (Keithley 2010) and a source meter (Keithley 2400). The Seebeck coefficient was measured by heating one resistor block while simultaneously measuring the generated temperature gradient (Δ*T*) and TE voltage (Δ*V*). The SCLC measurements were performed on the electron‐only and hole‐only devices with configurations of ITO/SnO_2_/THPs/PC_61_BM/Ag and ITO/PEDOT:PSS/THPs/PTAA/Au, respectively, in which a bias was scanned from 0 to 3 V with a step of 0.01 V. The obtained electrical characteristics were plotted in logarithmic coordinates with the vertical axis representing (*J × d*) in A cm^−1^ and the horizontal axis the electric field intensity (*E*) in V cm^−1^. The plots were then fit to calculate electron and hole mobility. XRD pattern data for 2*θ* values were collected with a Bruker AX D8 Advance diffractometer with nickel‐filtered Cu Kα radiation (*λ* = 1.5406 Å). GIWAXS experiments were carried out at the Shanghai Synchrotron Radiation Facility (SSRF). The samples were prepared on glass substrates and the data were obtained with an area CCD detector of 3072 × 3072 pixels resolution (225 mm × 225 mm) at beamline BL14B1. The monochromated energy of the X‐ray source was 10 keV, while the X‐ray wavelength was 1.2378 Å and the incidence angle was set at 0.3°. FESEM images were acquired on ZEISS Gemini 300 at an accelerating voltage of up to 30 kV. XPS analysis was conducted with X‐ray photoemission spectroscope (PHI5300). All the peaks were calibrated by C1*s*. TOF‐SIMS measurements were obtained by TOF.SIMS 5 (IONTOF). Oxygen‐ion sputtering was used for depth proﬁling with a focused Bi primary ion pulse for analysis. An area of (167 × 167 μm^2^) was characterized to ensure the uniformity of sampling. Thermal conductivity was obtained by the customer‐built scanning thermal microscopy (SThM) which integrated a thermal sensor with an atomic force microscopy platform (NFP‐3D Infinity, Oxford). GLA SThM probe (produced by Anasys Instruments) was assembled in Wheatstone bridge.

## Conflict of Interest

The authors declare no conflict of interest.

## Supporting information

Supplementary Material

## Data Availability

The data that support the findings of this study are available from the corresponding author upon reasonable request.
