# Supplementary material for: Efficient p‐Type Doping of Tin Halide Perovskite via Sequential Diffusion for Thermoelectrics
Source: Small Sci. 2022 Mar 6;2(6):2200004. doi: 10.1002/smsc.202200004 (PMC11935806; doi:10.1002/smsc.202200004)
Supplement: Supplementary file 1 — Supplementary Material [file SMSC-2-2200004-s001.pdf]

Supporting Information

Efficient p-Type Doping of Tin Halide Perovskite via Sequential Diffusion for Thermoelectrics

Ruisi Chen,[1] Yajie Yan,[1] Junhui Tang,[1] Huarong Zeng,[2] Qin Yao,[2] Lidong Chen[2] and Ziqi Liang[1]**

[*][1]Prof. Z. Liang, R. Chen, Y. Yan, J. Tang

Department of Materials Science, Fudan University

Shanghai 200433, China

Email: zqliang@fudan.edu.cn

[*][2]Dr. Q. Yao, Dr. H. Zeng, Prof. L. Chen

State Key Laboratory of High-Performance Ceramics and Superfine Microstructure, Shanghai Institute of Ceramics, Chinese Academy of Sciences

Shanghai 200050, China

E-mail: yaoqin@mail.sic.ac.cn

Results

Table S1. The optimal oxidation time and highest σ at different humidity

	**Relative Humidity (%)**	**T (°C)**	**Optimal Oxidation Time (min)**	**Highest σ (S/cm)**
**$FASnI_3$**	20	26	12	2.51± 0.14
	65	26	10	1.34 ± 0.21

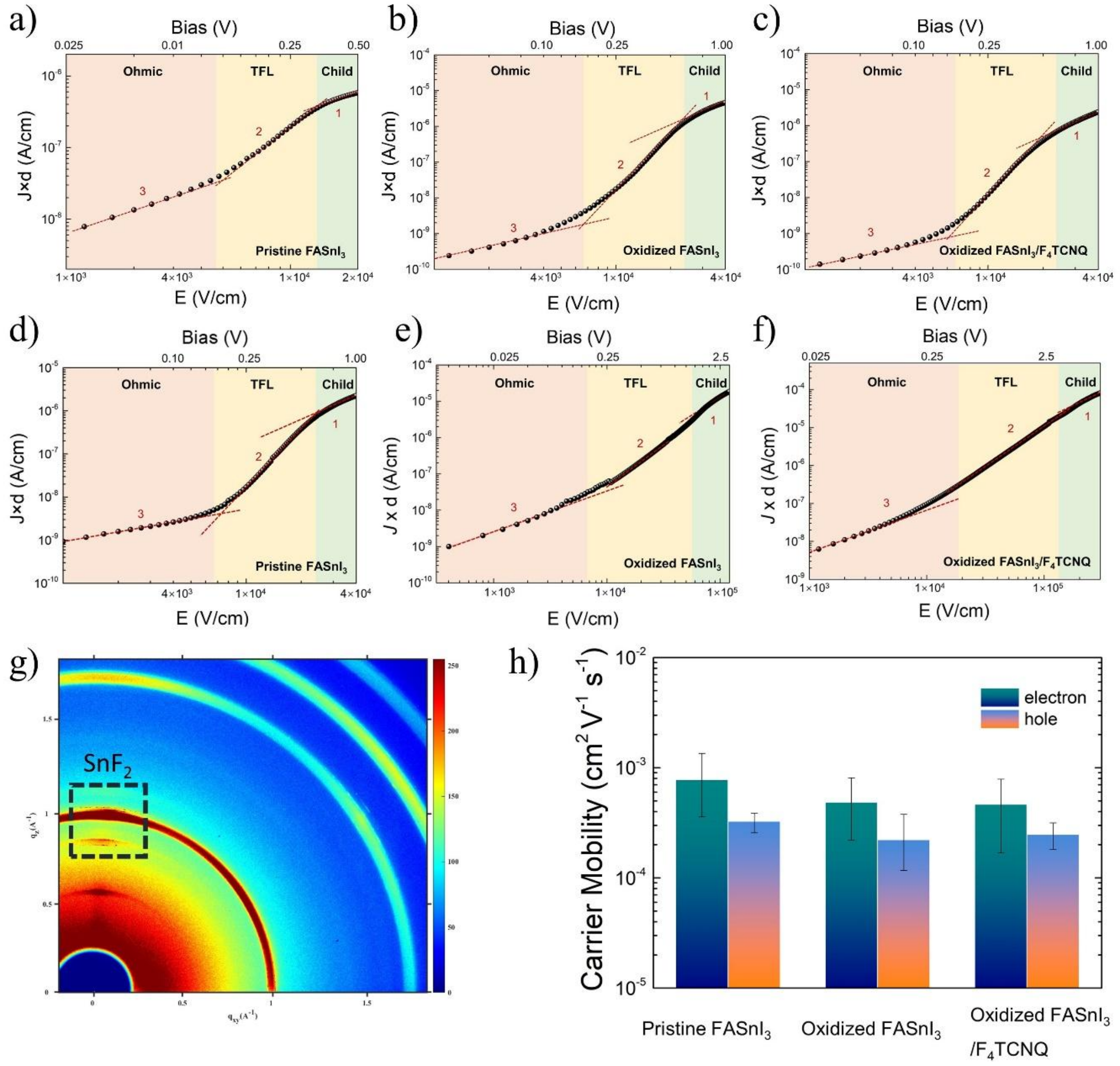

Figure S1. a‒f) Current vs. electric field characteristics of electron-only and hole-only devices via SCLC method. g) GIWAXS pattern of neat $FASnI_3$ film. h) A summary of electron and hole mobilities of pristine, oxidized and jointly-doped $FASnI_3$ thin films.

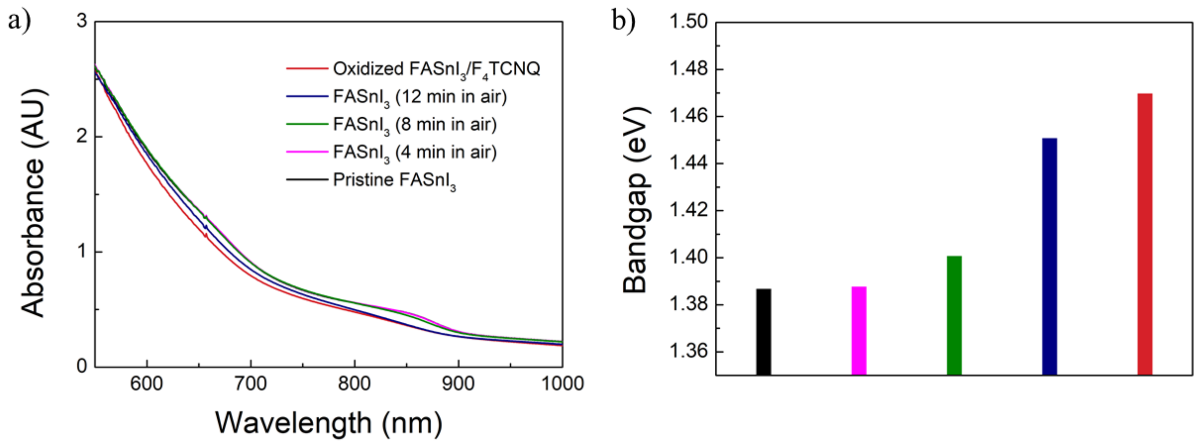

Figure S2. a) UV-vis spectra and b) the corresponding calculated bandgaps of neat and various treated $FASnI_3$ films.

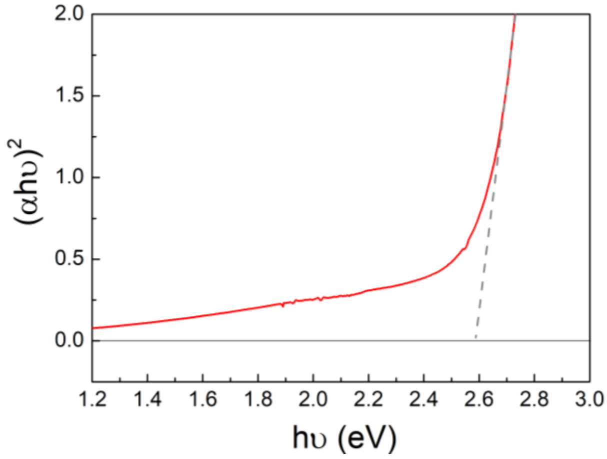

Figure S3. UV-vis spectra of SnI_4 film showing a bandgap of ~2.6 eV.

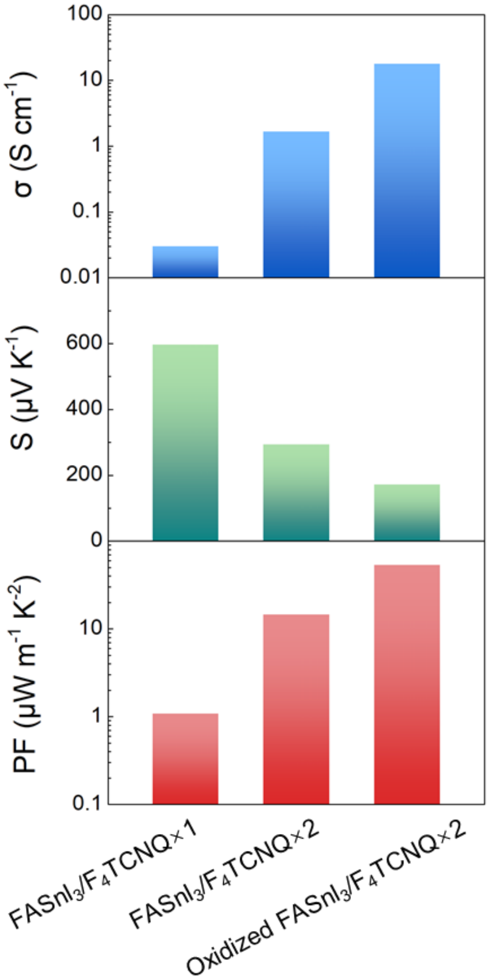

Figure S4. A sum of thermoelectric properties of neat $FASnI_3$ films with various numbers of F_4TCNQ layer and oxidized $FASnI_3$ with 2 layers of F_4TCNQ.

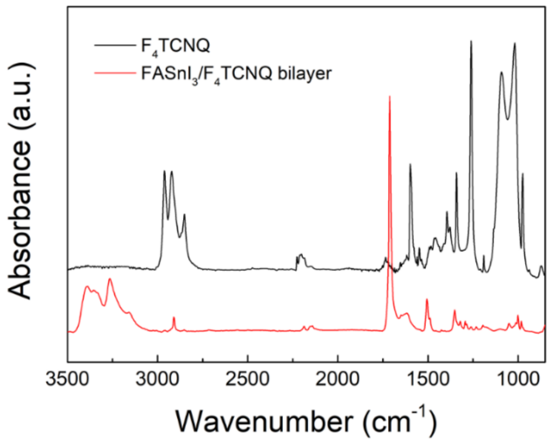

Figure S5. Full FTIR spectra of neat F_4TCNQ and $FASnI_3/F_4TCNQ$ bilayer films.

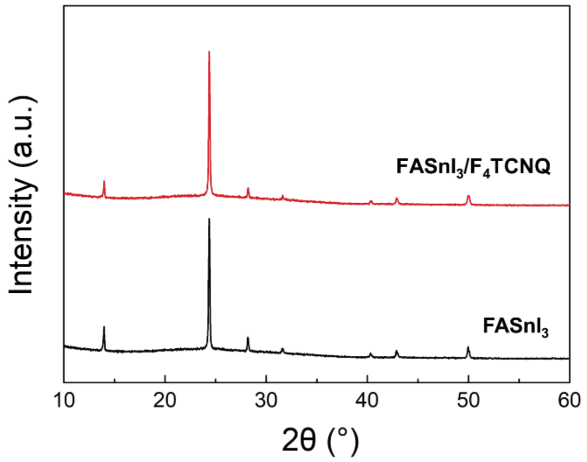

Figure S6. XRD patterns of $FASnI_3$ thin film w and w/o F_4TCNQ doping.

Thermal conductivity Scanning thermal microscopy (SThM) measurement

In this study, SThM is adopted to measure the thermal conductivity of $FASnI_3$ films on glass substrate. Since the heat tip is extremely thermosensitive and the resistance-temperature relationship is linear, the temperature change of the tip can be controlled and monitored by applying an electric current to the probe. Consequently, the change of ambient thermal resistance (TR) results in the variation of probe voltage. Here, the thermal tip-sample approach curve is used to calculate the thermal conductivity.[S1] When the tip abruptly upon contact, the thermal circumstance does not change. Before contact, the heat ($Q_{air,off}$) generated by the tip all dissipates into the air, while after contact, a part of the heat (Q_{sample}) diffuses into the sample, which can be described as:

$$\text{Off contact: } P_{off} = Q_{air,off} = A \cdot h \cdot \Delta T_{off} \qquad \text{(S1)}$$

$$\text{On contact: } P_{on} = Q_{air,on} + Q_{sample} = A \cdot h \cdot \Delta T_{on} + \frac{1}{R_{sample}} \cdot \Delta T_{on} \qquad \text{(S2)}$$

where the A is the superficial area of the tip, h is the heat transfer coefficient of the tip, R_{sample} is the sample thermal resistance, P_{off} and P_{on} are the heat power generated by the thermal

probe before and after contact, respectively. Based on the above model, R_{sample} can be deduced as following:

$$\frac{1}{R_{sample}} = \frac{P_{on}}{\Delta T_{on}} - \frac{P_{off}}{\Delta T_{off}} \tag{S3}$$

Here we chose polycrystalline $MAPbI_3$ film (κ = 0.3 W m^{-1} K^{-1}) and polypropylene (κ = 0.26 W m^{-1} K^{-1}) as the reference samples. Considering the hemispheric distribution of the temperature field and assuming the contact thermal resistance is consistent, the thermal conductivity of the samples can be resolved.

Reference

[S1] K. Xu, S. Ye, L. Lei, L. Meng, S. Hussain, Z. Zheng, H. Zeng, W. Ji, R. Xu, Z. Cheng, Dynamic interfacial mechanical-thermal characteristics of atomically thin two-dimensional crystals, Nanoscale 10 (2018) 13548−13554.
